# Gemcitabine-loaded chitosan nanoparticles enhanced apoptotic and ferroptotic response of gemcitabine treatment alone in the pancreatic cancer cells in vitro

**DOI:** 10.1007/s00210-024-03193-6

**Published:** 2024-06-17

**Authors:** Duygu Aydemir, Kıvılcım Öztürk, Fatma Betül Arslan, Sema Çalis, Nuriye Nuray Ulusu

**Affiliations:** 1 School of Medicine, Department of Medical Biochemistry, Koc University, Istanbul, Turkey; 2 Koç University Research Center for Translational Medicine (KUTTAM), Istanbul, Turkey; 3 Department of Pharmaceutical Technology, Faculty of Pharmacy, Hacettepe University, Ankara, Turkey; 4 Biochemistry Department, Koc University School of Medicine, Rumelifeneri Yolu, Sariyer, Istanbul, 34450 Turkey

**Keywords:** Gemcitabine, Chitosan, Oxidative stress, Apoptosis, Pancreatic cancer, Ferroptosis

## Abstract

Gemcitabine (GEM) is a first-line treatment for pancreatic ductal adenocarcinoma (PDAC) patients, causing side effects and poor overall survival. Eighty percent of patients often develop resistance rapidly to GEM. Developing therapeutic approaches and increasing sensitivity to gemcitabine in PDAC has become one of the challenges in cancer research. We synthesized GEM-loaded NPs prepared with a method that combines ultrasonication and ionotropic gelation to overcome GEM-related limitations in PDAC. CFPAC-1 cells were treated with increased concentrations of GEM, empty chitosan, and GEM-loaded NPs (0.66, 1.32, 2.64, 5.32 µg/ml) for up to 48 h. Empty chitosan NPs did not show toxicity on L929 cells. Antioxidant enzyme activities, including glucose 6-phosphate dehydrogenase (G6PD), 6-phosphogluconate dehydrogenase (6-PGD), glutathione reductase (GR), glutathione s-transferase (GST), and glutathione peroxidase (GPx), significantly reduced in GEM-loaded NPs compared to the GEM associated with increased oxidative stress, PPP, and glycolysis. Bcl-xL, NOXA/mcl-1, and Ca^2+^ levels significantly increased in GEM-loaded NP-administered cells compared to the GEM and control groups. In contrast, JNK, p38, STAT3, Akt, and CREB levels significantly decreased in the GEM-loaded NP group, addressing enhanced apoptotic response compared to the GEM alone. Increased ferroptosis activity in GEM-loaded NP-administered groups has been validated via decreased antioxidant enzyme activities, increased cytosolic Fe, Zn, Mg, and Mn levels, and reduced GPx activity compared to the GEM and control groups. For the first time in the literature, we showed biocompatible GEM-loaded NPs enhanced apoptotic and ferroptotic response in CFPAC-1 cells via downregulation of antioxidant, glycolysis, and PPP metabolism compared to the GEM alone.

**Graphical abstract:**

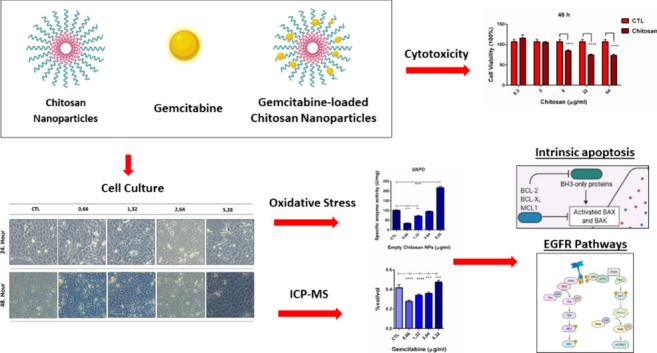

## Introduction

Pancreatic cancer is one of the most aggressive cancer types with a poor prognosis and a high mortality rate of almost 88%, with a 5-year survival rate of 12% (Halbrook et al. 2023). Pancreatic ductal adenocarcinoma (PDAC) accounts for 90% of all pancreatic cancer cases, and it is expected to become the second most common cancer type worldwide in the next decade (Sun et al. 2021). Gemcitabine (2′,2′-difluoro-2′-deoxycytidine, GEM) is a deoxycytidine analog with a highly hydrophilic structure, currently used in the clinics for the treatment of several types of human cancers, including breast, thyroid, colon, ovarian, non-small cell lung, bladder, and pancreatic cancers (Khaira et al. 2014). GEM is a first-line treatment for pancreatic cancer patients who are not eligible for surgery; however, it has side effects and poor overall survival. Patients often develop resistance rapidly to GEM because of the reduced cellular uptake and short blood circulation time (17 min). Continuous intravenous (IV) infusion of GEM is needed to achieve therapeutic concentration in plasma (Öztürk et al. 2017; Sun et al. 2021).

GEM resistance and effectiveness are mainly modulated with MAPK, PI3K/Akt, and NF-*κ*B pathways; anti-apoptotic proteins involved in intrinsic apoptosis, such as Bcl-xL, Mcl-1, and bcl-2; and tumor suppressor proteins KRAS and p53 (Hamacher et al. 2008; de Sousa Cavalcante and Monteiro 2014). Mutations in KRAS and p53 are the most common genetic alterations in PDAC associated with the upregulation of oxidative stress, pentose phosphate pathway (PPP), and glycolysis; in contrast, decreased apoptosis and ferroptosis lead to resistance and decreased tumor sensitivity against GEM (Yang et al. 2021). On the other hand, dysregulation of the glucose metabolism, PPP, oxidative phosphorylation (OXPHOS), glutamine metabolism, hypoxia, and oxidative stress metabolism are the main contributors to the chemoresistance, metastasis, and cancer progression in all types of cancers, including PDAC (Barrera et al. 2021), because substrates for the bioenergetic and metabolic pathways required for tumor growth are glutamine, glucose, lactate, pyruvate, acetate, free fatty acids, and β-hydroxybutyrate synthesized excessively via upregulation of glycolysis, PPP, and oxidative stress metabolisms (Sonveaux et al. 2008). Therefore, research currently focuses on the therapy approaches for PDAC to develop new drugs and overcome gemcitabine-related limitations (Fujiwara-Tani et al. 2022).

Nanotechnology-based drug delivery systems are promising therapeutic approaches to overcome conventional chemotherapy treatments’ limitations (Aydemir et al. 2019a, b, 2020a). Using polymers to create nanotechnology has gained tremendous interest in the pharmaceutical industry and the development of novel drug delivery systems. Natural biodegradable polymer chitosan, derived from crustacean shells, is widely used in oral drug delivery, nasal drug delivery, pulmonary delivery, and tumor targeting studies due to their unique properties like permeation enhancing, non-toxicity, and mucoadhesion (Quiñones et al. 2018; Zhang et al. 2019). Chitosan has superior properties, including high biocompatibility, biodegradability, low toxicity, site-specific drug targeting, anti-microbial and hemostatic activity, ease of modification, and manufacturing that distinguishes chitosan from other polymers for nanoformulations. On the other hand, physicochemical properties can be modified to enhance the efficiency of drug delivery and release, for instance, pH sensitivity, intestinal solubility, tight junction passing, and permeabilization of the cell membrane can be modulated via chitosan modifications (Garg et al. 2019). Therefore, chitosan nanoparticles are considered promising tools for drug delivery systems with reduced therapeutic doses and side effects (Ozturk et al. 2020a). In this study, we synthesized gemcitabine-loaded chitosan nanoparticles and evaluated their impact on apoptotic pathways, ferroptosis, oxidative stress metabolism, PPP, and glycolysis compared with gemcitabine alone in CFPAC-1 cell line first time in the literature.

## Materials and methods

### Materials

SUPRAPUR® nitric acid (HNO_3_) and cell signaling multiplex assays #48-681MAG and #48-683MAG were purchased from Merck (Darmstadt, Germany). Chitosan (Protasan™ UP CL113) was obtained from NovaMatrix® (Sandvika, Norway). Dulbecco’s modified Eagle’s medium (DMEM) and fetal bovine serum (FBS) were purchased from Biochrom (Berlin, Germany). Trypsin-EDTA was obtained from PAA (Pasching, Austria). All other chemicals were purchased from Sigma-Aldrich Co. (St. Louis, MO, USA). The CFPAC-1 cell line was purchased from ATCC.

### Preparation and characterization of chitosan nanoparticles

Chitosan NPs were prepared using ionotropic gelation and ultrasonication techniques (Malatesta et al. 2015; Ozturk et al. 2020b). Chitosan NPs were prepared, as reported in our previous study (Ozturk et al. 2020b). Triphenyl phosphate (TPP) solution (0.75 mg/ml) was added into CS solution (1 mg/ml) under ultrasonication (sonication amplitude 20 W, sonication time 10 min) and magnetic stirring (550 rpm—10 min). The suspension was centrifuged at 12,500 rpm for 25 min in a glycerol bed to collect CS NPs and remove free TPP and CS molecules. Loaded NPs were prepared using the abovementioned method after dissolving the GEM in an aqueous CS phase (0.5 mg/ml). Synthesized NPs were characterized, and particle size, particle size distribution, surface charge, morphology, encapsulation efficiency, in vitro drug release, stability, cytotoxicity on the CFPAC-1 cell line, and cellular uptake of chitosan NPs were reported in our previous work (Ozturk et al. 2020b).

### Evaluation of polymer toxicity

Mouse fibroblast cell line L929 (American Type Culture Collection, LGC Promochem, Rockville, MD, USA), recommended by the US Pharmacopeial Convention (USP 42), was used to evaluate the cytotoxic effects of the chitosan NPs. L929 cells were cultured in DMEM supplemented with 10% FBS and 1% streptomycin-penicillin in a 37 °C humidified incubator with 5% CO_2_. Toxicity of the blank chitosan NPs was determined using the modified 3-(4,5-dimethyl- thiazole-2-yl)-2,5-diphenyltetrazolium bromide (MTT) assay as reported previously (Ozturk et al. 2020b). L929 cells were seeded in 96-well microplates (15 × 103 cells/well) in a 50 µl cell culture medium and incubated for 24 h. After redispersion in a serum-free culture medium, NPs were diluted to various concentrations with a serum-containing medium and added to the wells along with vehicle solvent-treated wells as controls. Cytotoxicity assays were performed in triplicates. Following 24 h and 48 h of incubation, 25 µl MTT (5 mg/ml) solution was added to each well, and the plates were further incubated for 4 h to allow formazan crystals to be formed by viable cells. Then, the cells were lysed (23% SDS in 80 µl 45% DMF lysis solution) during overnight incubation. The absorbance of each well was read at 570 nm using a microplate reader (Molecular Devices Corporation, Sunnyvale, CA, USA). Cell viability was calculated as a percentage change compared to the OD values obtained from untreated control cells.

### Evaluation of nanoparticle and gemcitabine toxicities on CFPAC-1 cell line

CFPAC-1 cells were cultured in DMEM supplemented with 10% FBS and 1% pen-strep and seeded in 25 cm^2^ flasks to expand after counting as described before (Otahal et al. 2020). When cells reached 70% confluency, they were treated with increased concentrations (0.66, 1.32, 2.64, 5.28 µg/ml) of gemcitabine-loaded chitosan NPs consisting of 6.25, 12.5, 25, 50 µg/ml chitosan respectively, gemcitabine alone (0.66, 1.32, 2.64, 5.28 µg/ml), and empty chitosan NPs (6.25, 12.5, 25, 50 µg/ml) for 48 h in a 37 °C humidified incubator with 5% CO_2_.

### Morphological evaluation of CFPAC-1 cells and preparation of cell lysates

Cells were observed every 24 h under the light microscope to evaluate morphological changes indicating apoptosis or ferroptosis upon NPs and gemcitabine treatment. After 48 h of treatment with either NPs or gemcitabine alone, cell media was aspirated, and cells were washed with ice-cold and sterile PBS. Then, cells were collected in 1 ml ice-cold PBS by scraping off and centrifuged for 2 min at 4 °C at 33,800 × *g*. The supernatant was removed, and pellets were suspended in the ice-cold sodium phosphate buffer (pH 7.4) supplemented with a protease inhibitor cocktail. Samples were stored at −80 °C until further experiments were carried out.

### Measurement of protein concentration

According to the kit instructions, the soluble protein concentration in each cell lysate was evaluated via BCA assay using albumin as standard.

### Evaluation of pentose phosphate pathway enzymes

G6PD and 6-PGD enzyme activities were evaluated as described by Aydemir et al. previously by monitoring NADPH production for 60 s at 340 nm and 37 °C via LKB Ultraspec Plus spectrophotometer (4054 UV/visible, Biochrom Ltd., Cambridge, UK) (Aydemir et al. 2018, 2019c).

### Evaluation of the oxidative stress metabolism

Glutathione reductase (GR) enzyme activity in the cell lysates was measured using NADPH and oxidized glutathione (GSSG) as substrates at 340 nm for 60 s at 37 °C as described in detail by Aydemir et al. (Aydemir et al. 2019d). On the other hand, reduced glutathione (GSH) and CDNB were used as substrates to measure GST enzyme activity for 30 s at 340 nm and 37 °C, as described previously. Both enzyme activities were measured using an LKB Ultraspec Plus spectrophotometer (4054 UV/visible, Biochrom Ltd., Cambridge, UK) (Aydemir et al. 2019d).

### Evaluation of total glutathione peroxidase (GPx) enzyme activity

Twenty microliter of cell lysate was added into the reaction mixture containing 100 mmol/l Na_2_PO_4_ (pH 7.4), 200 mmol/l EDTA, 400 mmol/l sodium azide, GR enzyme (10 U/ml), 2 mmol/l NADPH, 100 mmol/l GSH, and distilled water. The reaction mixture was incubated at 37 °C for 10 min, and then, 5 µl of 10 mmol/l H_2_O_2_ was added to the reaction mixture. As described previously, the system’s optical density (OD) decrease was evaluated at 340 nm at room temperature for 30 s.

### Evaluation of intrinsic and extrinsic apoptotic pathways via Luminex multiplex technology

Luminex multiplex technology using magnetic beads was used to evaluate intrinsic apoptotic pathways via Bcl-xL, Mcl-1, BCL-2, and NOXA/Mcl-1 quantification. Furthermore, extrinsic apoptotic and cancer signaling pathways were assessed via quantification of total Erk/MAPK½, Akt, STAT3, JNK, p70 S6 kinase, NFκB, STAT5A/B, CREB, and p38 proteins in the cell lysates.

### Microwave digestion

Cell lysates were dissolved with the acidic treatment in the microwave digestion system (Milestone START D). One hundred microliter cell lysate was dissolved in 10 ml of 65% HNO_3_ of EMSURE® and SUPRAPUR® grades. As reported, 100–200 µl of the serum samples was used for ICP-MS (Aydemir et al. 2020b).

### Evaluation of trace element and mineral levels via ICP-MS

After microwave digestion, cell lysate samples were diluted in the ultrapure water (ddH_2_O) at 1/10 dilution. Trace and mineral elements in cell lysates were measured by Agilent 7700x ICP-MS (Agilent Technologies Inc., Tokyo, Japan). MassHunter Work Station software creates the batch and analyzes the data. The peak pattern was drawn from 3 points, and the measurements were replicated five times (Aydemir et al. 2020b).

### Statistical analysis

GraphPad Prism and MILLIPLEX® Analyst 5.1 software were used to analyze the data. MILLIPLEX® Analyst 5.1 software was used to analyze MILLIPLEX® MAP kits. The rest of the data were analyzed using GraphPad Prism 9 software. Datasets were analyzed using two-way ANOVA followed by Tukey’s post hoc test for multiple comparisons. All data were represented as the mean ± standard deviation (SD), and *p* ≤ 0.05 was considered significant for all analyses.

## Results

### Characterization of nanoparticles

It has been found that the size of blank chitosan nanoparticles was 168.9 ± 7.23 nm with a 0.232 ± 0.01 polydispersity index. The surface charge of nanoparticles was 28.07 ± 2.50 mV. The encapsulation efficiency of gemcitabine obtained with this formulation was 35.18 ± 2.33%. In vitro drug release was evaluated in serum-containing cell culture medium at pH 6.0 and 7.5, serum-free cell culture medium at pH 6.0 and 7.5, and phosphate-buffered saline at pH 7.4. pH 6.0 mimics the pH of the cell culture medium when cells reach 85% confluency. The cumulative gemcitabine releases from chitosan nanoparticles were found as 42.9 ± 2.5%, 80.7 ± 5%, 68.7 ± 5.1%, 50.85 ± 2.6%, and 39.85 ± 0.65% within 24 h in pH 7.4 PBS, serum-free cell culture medium pH 6.0, serum-free cell culture medium pH 7.5, serum-containing cell culture medium pH 6.0, and serum-containing cell culture medium pH 7.5 within 24 h, respectively. A new method was developed to overcome the aggregation problem, especially for MTT assay. Gemcitabine-loaded chitosan nanoparticles (NPs) showed lower cell viability than gemcitabine solution at the highest concentration, 41.92 ± 2.01% and 26.23 ± 2.78%, respectively (Ozturk et al. 2020b). Because of the modified MTT method assay, blank chitosan nanoparticles did not have a significant cytotoxic effect on CFPAC-1 cells since the number of viable cells was 95–80% at 48 h. A stability assay of the chitosan nanoparticles has been performed using particle size, PDI, and zeta potential following the re-dispersion of nanoparticles in the cell media. Serum-containing and serum-free media conditions cause chitosan nanoparticles to have more tendency to aggregate; however, particle size stayed smaller than 10 µM at pH 7.4. Zeta potential values of the chitosan nanoparticles shifted from 0 to −5 mV. On the other hand, blank chitosan nanoparticles were highly stable at 4 °C and 25 °C for storage in which particle size did not dramatically change in the distilled water and serum-containing media (Ozturk et al. 2020a). Ozturk et al. recommended a pH value of more than 6.0 and a short incubation time of the blank chitosan and GEM-loaded chitosan nanoparticles to avoid aggregation of nanoparticles. Also, they have reported that adding glycerol did not prevent aggregation of the chitosan nanoparticles at different temperatures (25 and 37 °C) and pH values (6 and 7.4) (Ozturk et al. 2020a) (Table 1).

**Table 1:** The physicochemical properties of the chitosan nanoparticles are represented in the table. Encapsulation efficiency is represented by 10 min of magnetic stirring time. Data is represented by triplicates of the mean ± SD (Ozturk et al. 2020a)

Particle size (nm)	PDI	Zeta potential (mV)	Encapsulation efficiency (%)
168.9 ± 7.23	0.232 ± 0.01	28.07 ± 2.5	35.18 ± 233

### Evaluation of polymer toxicity

**Fig. 1:**
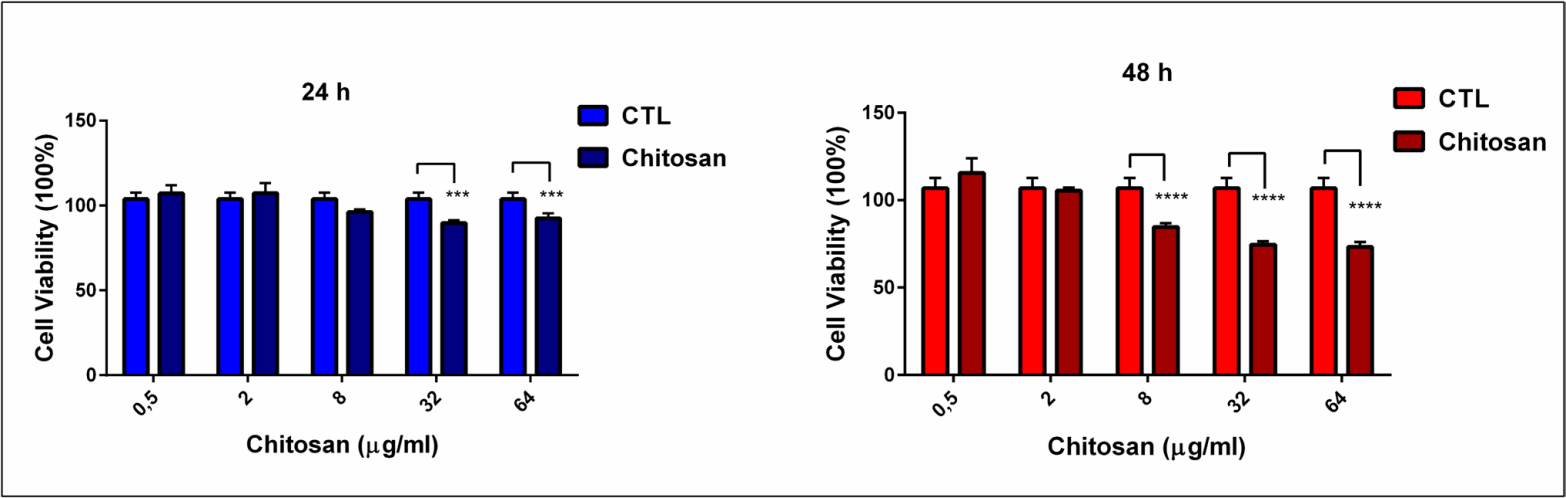
Cell viability of L929 cells was measured after treatment with an increased concentration of blank chitosan nanoparticles (NPs) (0.5, 2, 8, 32, 64 µg/ml). Each experiment was performed three times, and data are represented as the mean of standard deviation (SD). Datasets were analyzed using two-way ANOVA followed by Tukey’s post hoc test for multiple comparisons. All data were represented as the mean ± standard deviation (SD), and *p* ≤ 0.05 was considered significant for all analyses. Untreated cells were used as the control group (CTL). Viability values at 24 and 48 h for the highest polymer concentration (64 µg/ml) treated cells were approximately 90% and 70%, respectively. Notes: *p < 0.05, **p < 0.001, and ***p < 0.000

The viability of L929 cells after treatment with increasing concentrations of blank chitosan nanoparticles was determined using the modified MTT assay. Viability values at 24 and 48 h for the highest polymer concentration (64 µg/ml) treated cells were approximately 90% and 70%, respectively (Fig. 1).

### Morphological changes of CFPAC-1 cells upon loaded and empty nanoparticle treatment

**Fig. 2:**
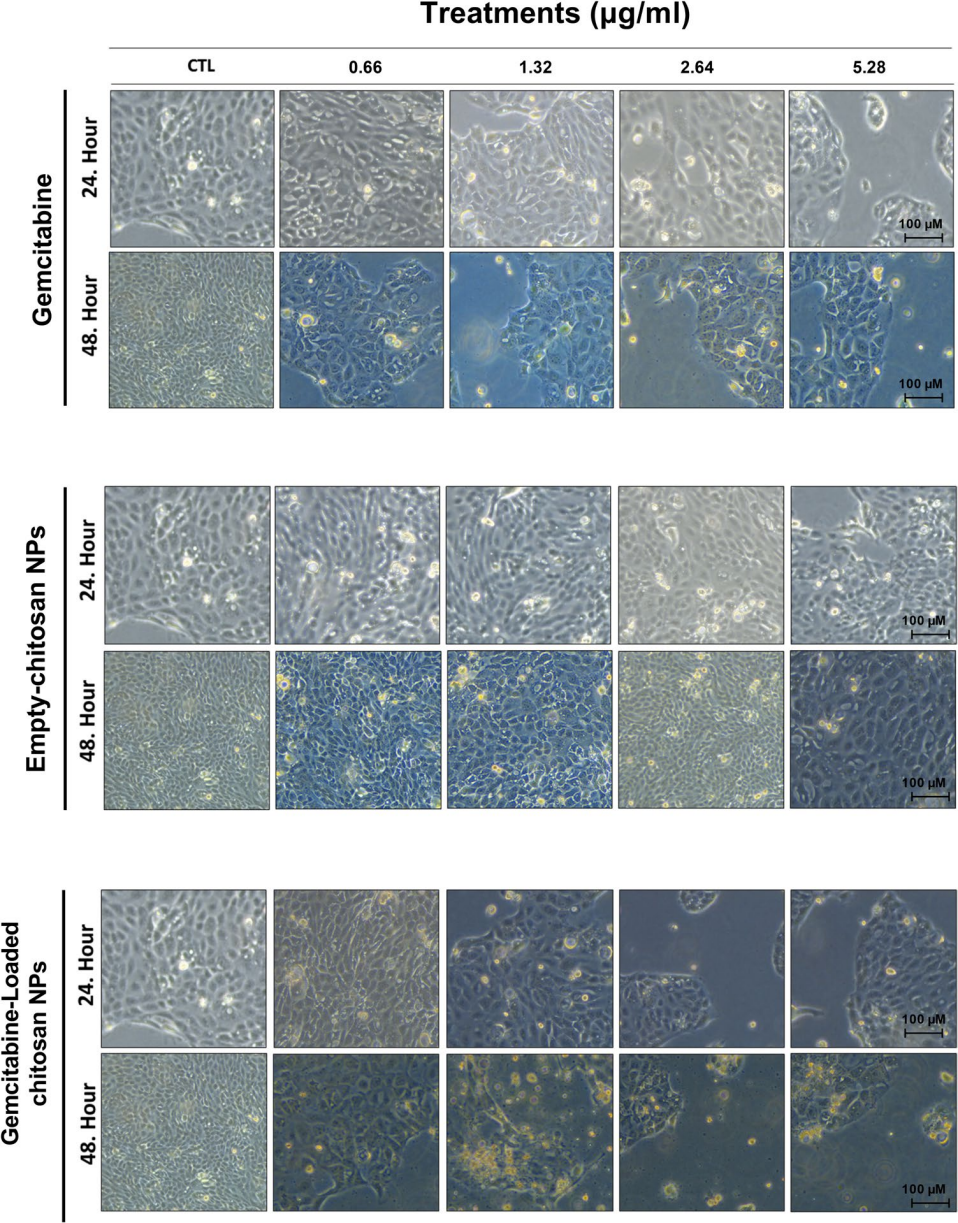
CFPAC-1 cells were treated with an increased concentration of empty chitosan nanoparticles (NPs), gemcitabine alone, and gemcitabine-loaded nanoparticles (NPs) for 48 h. Cell morphological changes were observed under a light microscope daily, and pictures were taken every 24 h. Each experiment was carried triplicate, and a picture of the same control group was used to compare to treated cells

Upon treatment with gemcitabine-loaded NPs and gemcitabine alone, morphological changes were investigated every 24 h under a light microscope using a CFPAC-1 cell line. Empty chitosan nanoparticles supported cell proliferation up to 25 µg/ml concentration (corresponding polymer concentration to 2.64 µg/ml gemcitabine-loaded NPs) compared to the control cells (Fig. 2). Gemcitabine reduced cell proliferation at each concentration compared to the control; however, any apoptotic markers have not been observed according to the light microscopy images (Fig. 2). Addition to the reduced cell proliferation, gemcitabine-loaded NPs induced apoptosis in the CFPAC-1 cells compared to the control and gemcitabine alone according to the data (Fig. 2).

### Gemcitabine-loaded NPs reduced cell proliferation more effectively than gemcitabine alone

**Fig. 3:**
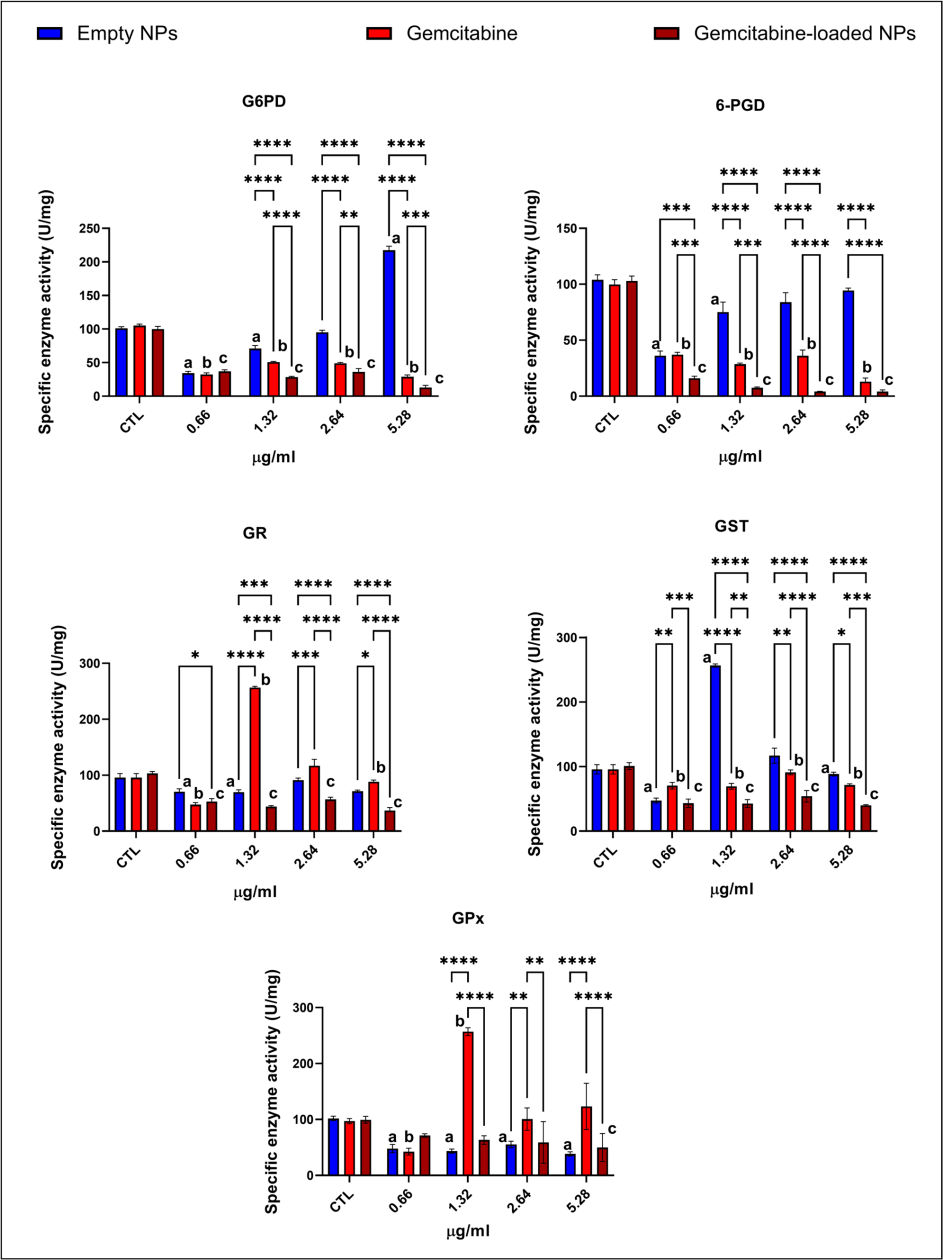
Cell proliferation and oxidative stress metabolism were evaluated via glucose 6-phosphate dehydrogenase (G6PD), 6-phosphoglucanate dehydrogenase (6-PGD), glutathione reductase (GR), glutathione s-transferase (GST), and glutathione peroxidase (GPx) enzyme. The cell proliferation rate was evaluated by measuring G6PD and 6-PGD enzyme activities involved in the pentose phosphate pathway (PPP) and glycolysis. The oxidative stress metabolism of cells was evaluated by measuring GR, GST, and GPx enzymes. Enzyme activities were normalized to the control group (100%). Each experiment was performed three times, and data are represented as the mean of standard deviation (SD). Untreated cells were used as control groups (CTL). Datasets were analyzed by the two-way ANOVA followed by Tukey’s post hoc test for multiple comparisons. All data were represented as the mean ± standard deviation (SD), and *p* ≤ 0.05 was considered significant for all analyses. ^a^Significantly different than the empty chitosan NP control group. ^b^Significantly different than the gemcitabine treatment control group. ^c^Significantly different than the gemcitabine-loaded NP control group.  Notes: *p  < 0.05, **p  < 0.01, ***p  < 0.001, ****p  < 0.0001

All enzyme activities normalized against the control of each group. G6PD and 6-PGD enzyme activities significantly reduced when treated with lower concentrations of 0.66–1.32 µg/ml of empty chitosan NPs compared to the control. On the other hand, administration with 25 and 50 µg/ml empty chitosan nanoparticles (corresponding polymer concentration to 2.64 µg/ml and 5.28 µg/ml gemcitabine-loaded NPs) significantly increased G6PD and 6-PGD activities. Gemcitabine treatment reduces indicated enzyme activities by 50% compared to the control; on the other hand, gemcitabine-loaded NPs significantly reduced both enzyme activities compared to the gemcitabine alone (Fig. 3).

### Gemcitabine-loaded NPs significantly reduced the antioxidant capacity of the CFPAC-1 cell line

GR, GST, and total GPx enzyme activities were measured in each treatment group to evaluate the antioxidant status of the CFPAC-1 cells. GR enzyme activity increased in the gemcitabine-treated groups (1.32, 2.64, 5.28 µg/ml) compared to the control cells. On the other hand, gemcitabine-loaded NPs significantly reduced GR activity compared to the control, gemcitabine, and empty chitosan NP-treated groups at each concentration (Fig. 3). Gemcitabine and gemcitabine-loaded NPs significantly reduced GST activity compared to the control groups; however, this decrease was higher in gemcitabine-loaded NP-treated groups (Fig. 3). GPx activity significantly reduced in gemcitabine-loaded NP-treated groups compared to the gemcitabine alone. This decrease was also observed compared to the control groups; however, this change was only significant in the 5.28 µg/ml group (Fig. 3).

### Gemcitabine-loaded NPs activated pro-apoptotic proteins and inhibited cell survival pathways addressing enhanced apoptosis

**Fig. 4:**
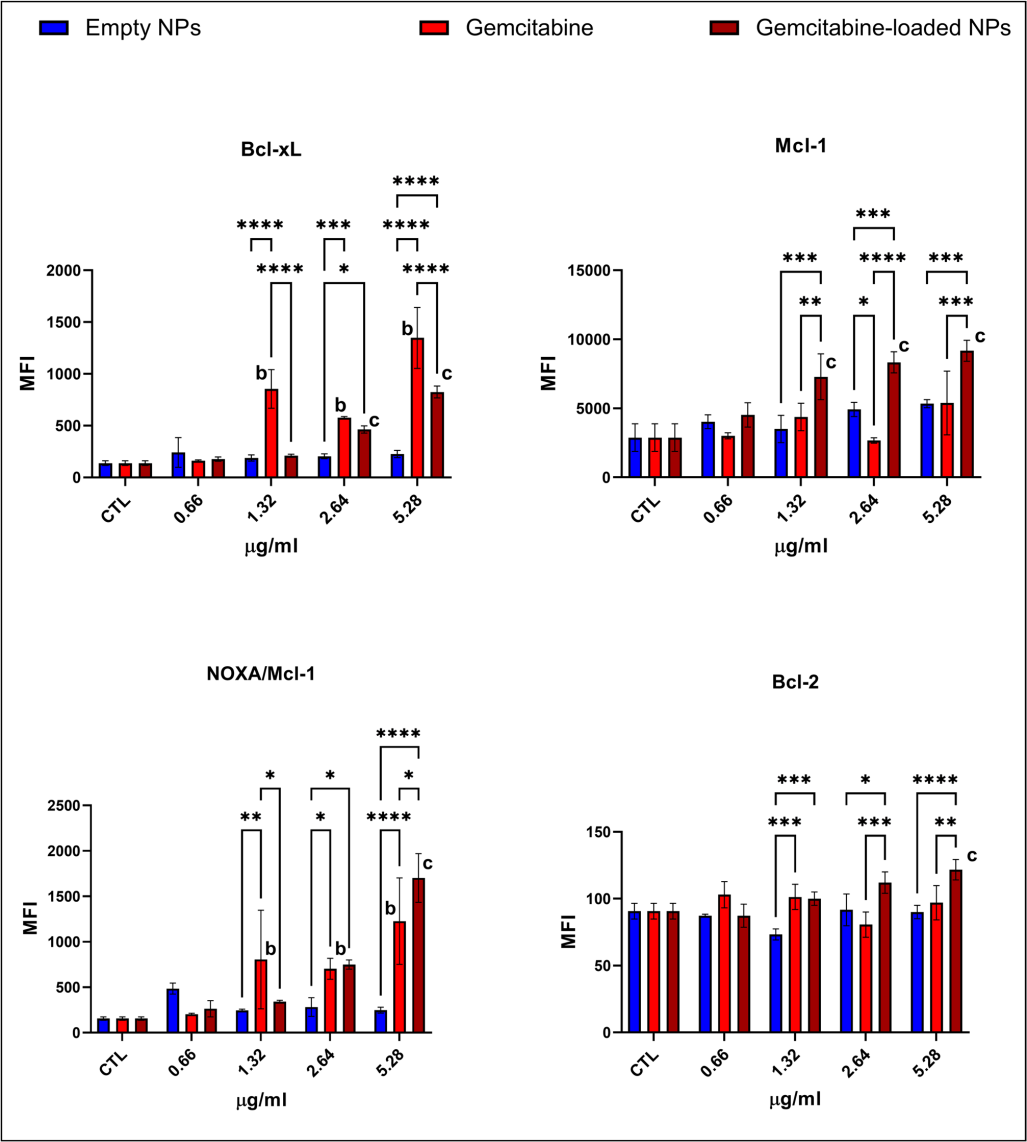
NOXA/mcl-1, bcl-2, bcl-xL, and mcl-1 proteins regulating intrinsic apoptosis were measured via Luminex multiplex technology using magnetic beads via MAGPIX® System. Measurement was represented as mean fluorescence intensity (MFI) calculated by MILLIPLEX® Analyst 5.1 software. Each experiment was performed three times, and data are represented as the mean of standard deviation (SD). Untreated cells were used as control groups (CTL). Datasets were analyzed by the two-way ANOVA followed by Tukey’s post hoc test for multiple comparisons. All data were represented as the mean ± standard deviation (SD), and *p* ≤ 0.05 was considered significant for all analyses. ^a^Significantly different than the empty chitosan NP control group. ^b^Significantly different than the gemcitabine treatment control group. ^c^Significantly different than the gemcitabine-loaded NP control group. Notes: *p < 0.05, **p < 0.01, ***p < 0.001, ****p < 0.0001

**Fig. 5:**
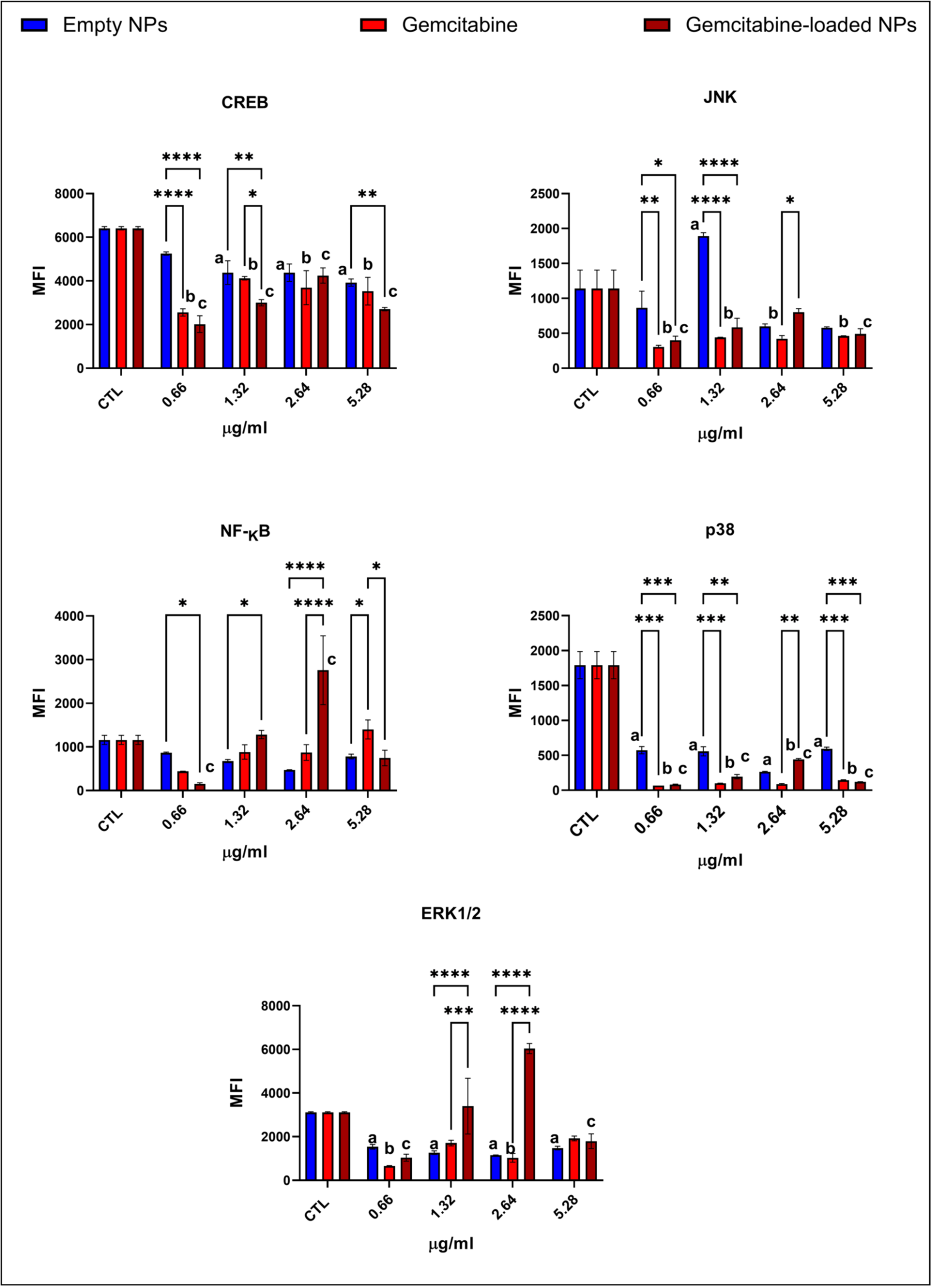
Oncogenic signaling pathways Akt, STAT3, STAT5, and p70 involved in the cell survival were evaluated via Luminex multiplex technology using magnetic beads via MAGPIX® System. Measurement was represented as mean fluorescence intensity (MFI) calculated by MILLIPLEX® Analyst 5.1 software. Each experiment was performed three times, and data are represented as the mean of standard deviation (SD). Untreated cells were used as control groups (CTL). Datasets were analyzed by the two-way ANOVA followed by Tukey’s post hoc test for multiple comparisons. All data were represented as the mean ± standard deviation (SD), and *p* ≤ 0.05 was considered significant for all analyses. ^**a**^ significantly different than the empty chitosan NPs control group, ^**b**^ significantly different than the gemcitabine-treatment control group, ^**c**^ significantly different than the gemcitabine-loaded NPs control group. Notes: *p < 0.05, **p < 0.01, ***p < 0.001, ****p < 0.0001

**Fig. 6:**
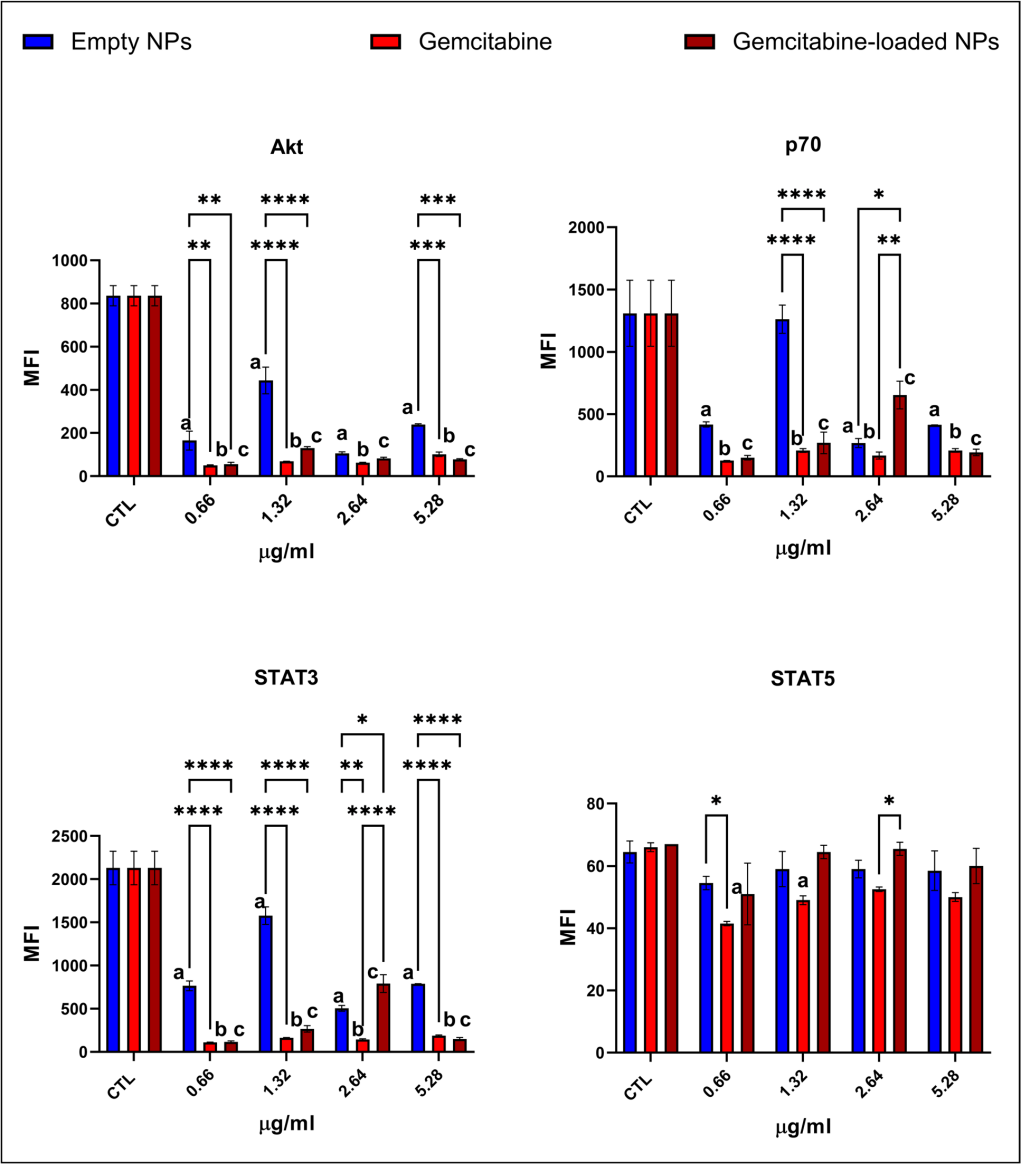
ERK1/2, p38, JNK, CREB, and NF-KB proteins are involved in EGFR signaling, intrinsic apoptosis, cell survival, oxidative stress, and extrinsic apoptosis. Indicated protein levels were measured via Luminex multiplex technology using magnetic beads via MAGPIX® System. Measurement was represented as mean fluorescence intensity (MFI) calculated by MILLIPLEX® Analyst 5.1 software. Untreated cells were used as control groups (CTL). Datasets were analyzed by the two-way ANOVA followed by Tukey’s post hoc test for multiple comparisons. All data were represented as the mean ± standard deviation (SD), and *p* ≤ 0.05 was considered significant for all analyses. ^a^Significantly different than the empty chitosan NP control group. ^b^Significantly different than the gemcitabine treatment control group. ^c^Significantly different than the gemcitabine-loaded NP control group.  Notes: *p < 0.05, **p < 0.01, ***p < 0.001, ****p < 0.0001

**Fig. 7:**
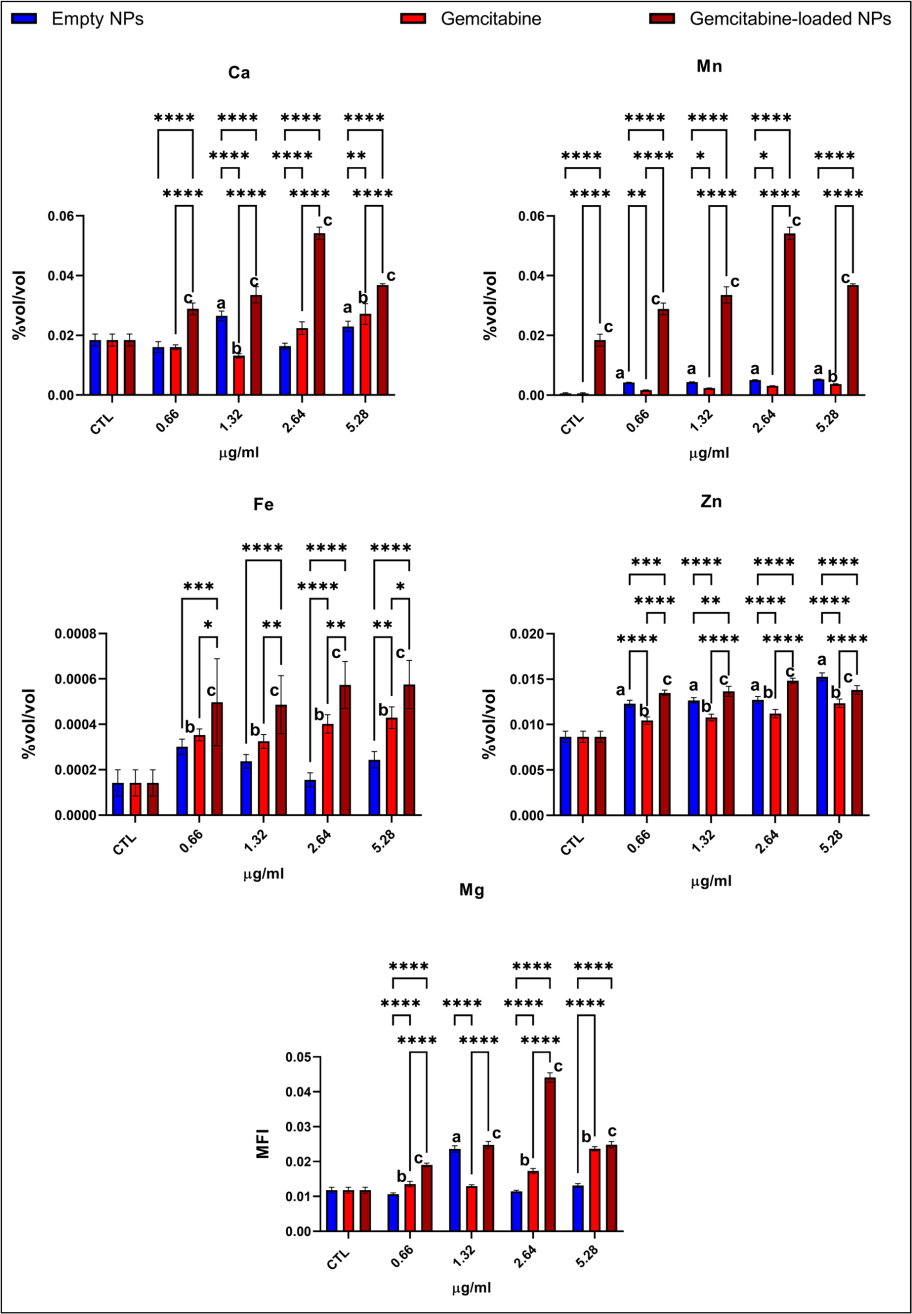
Trace element and mineral levels, including Ca, Zn, Fe, and Mn, involved in ferroptosis, apoptosis, and oxidative stress metabolism were evaluated via ICP-MS following microwave acidic digestion. Untreated cells were used as control groups (CTL). Datasets were analyzed by the two-way ANOVA followed by Tukey’s post hoc test for multiple comparisons. All data were represented as the mean ± standard deviation (SD), and *p* ≤ 0.05 was considered significant for all analyses. ^a^Significantly different than the empty chitosan NP control group. ^b^Significantly different than the gemcitabine treatment control group. ^c^Significantly different than the gemcitabine-loaded NP control group. Notes: *p < 0.05, **p < 0.01, ***p < 0.001, ****p < 0.0001

Anti-apoptotic proteins Bcl-xL and NOXA/mcl-1 levels were lower in the cells treated with the gemcitabine-loaded NPs than in the gemcitabine-treated cells except the 0.66 µg/ml group (Fig. 4). On the other hand, Bcl-2 and Mcl-1 levels were slightly higher in gemcitabine-loaded NP-administered cells compared to the gemcitabine-treated cells (2.64, 5.32 µg/ml) (Fig. 4). CREB, JNK, p38, Akt, p70, and STAT3 levels are significantly lower in the gemcitabine and gemcitabine-loaded NP-treated groups at each concentration (Figs. 5 and 6). On the other hand, cytosolic Ca levels increased dramatically in the gemcitabine-loaded NP-administered cells compared to the control and gemcitabine-treated cells (Fig. 7).

#### Gemcitabine-loaded NPs enhanced ferroptosis via the decreased antioxidant status of the cell

G6PD, 6PGD, GR, GST, and GPx activities significantly decreased in the gemcitabine-loaded NP-administered cells compared to the gemcitabine-treated and control cells (Fig. 3). NF-KB and ERK1/2 pathways significantly upregulated in gemcitabine-loaded NP-administered cells compared to the control and gemcitabine-treated cells at 1.32 and 2.64 µg/ml concentrations (Fig. 5). Intracellular Fe, Mg, Zn, and Mn levels significantly increased gemcitabine-loaded NP-administered cells compared to the gemcitabine-treated and control cells (Fig. 7).

## Discussion

Pancreatic cancer is one of the most aggressive cancer types, with a poor prognosis and a high mortality rate of almost 88%, and 5-year survival is 12% according to Globocan Statistics 2023. GEM-based chemotherapy is a standard therapy for patients with pancreatic cancer despite adverse effects and poor overall survival. Eighty percent of all pancreatic cancer patients develop chemoresistance against GEM in locally advanced or metastatic phases, and the median survival of those patients is 6 months or less (Ju et al. 2015; Fujiwara-Tani et al. 2022). Therefore, developing therapeutic approaches and increasing sensitivity to gemcitabine in PDAC has become one of the most significant challenges in cancer research, and various studies focus on the prolonged lifetime of patients with PDAC (Ju et al. 2015). Chitosan nanoparticles attract more attention daily because of their advantageous properties, including anti-microbial effects, bioavailability, biodegradability, good blood flow characteristics, and improving drug efficacy (Singla and Chawla 2010; Nilsen-Nygaard et al. 2015; Bellich et al. 2016). Therefore, in this study, we synthesized gemcitabine-loaded NPs to evaluate their anti-cancer properties compared to gemcitabine alone.

*KRAS* and *p53* are the most common genetic alterations in PDAC associated with altered glycolysis, PPP, and oxidative stress metabolism leading to poor prognosis, chemoresistance, metastasis, and cancer progression (Liu et al. 2019). Therefore, we have chosen the CFPAC-1 cell line harboring KRAS and p53 mutations in this study. Previously, we have reported the optimization of the synthesis of gemcitabine-loaded chitosan NPs and troubleshooting in the MTT assay procedure, a concerning problem of the aggregation of chitosan nanoparticles (Ozturk et al. 2020b). In this study, blank nanoparticles were evaluated employing polymer safety using L929 healthy fibroblast cells at a polymer concentration range of 0.5–64 µg/ml, and cell viability was ≥70% after 48 h incubation with these particles (Fig. 1). Viability values of 70% and over are considered non-cytotoxic by ISO 10993-5 [28]. The size of the blank chitosan nanoparticles was 168.9 ± 7.23 nm with a 0.232 ± 0.01 polydispersity index. The surface charge of nanoparticles was 28.07 ± 2.50 mV. The encapsulation efficiency of gemcitabine obtained with this formulation was 35.18 ± 2.33%. In vitro drug release was evaluated in serum-containing cell culture medium at pH 6.0 and 7.5, serum-free cell culture medium at pH 6.0 and 7.5, and phosphate-buffered saline at pH 7.4. pH 6.0 mimics the pH of the cell culture medium when cells reach 85% confluency. The cumulative gemcitabine releases from chitosan nanoparticles were found as 42.9 ± 2.5%, 80.7 ± 5%, 68.7 ± 5.1%, 50.85 ± 2.6%, and 39.85 ± 0.65% within 24 h in pH 7.4 PBS, serum-free cell culture medium pH 6.0, serum-free cell culture medium pH 7.5, serum-containing cell culture medium pH 6.0, and serum-containing cell culture medium pH 7.5 within 24 h, respectively. A new method was developed to overcome the aggregation problem, especially for MTT assay (Ozturk et al. 2020b). Because of the modified MTT method assay, blank chitosan nanoparticles did not have a significant cytotoxic effect on CFPAC-1 cells since the number of viable cells was 95–80% at 48 h. Gemcitabine-loaded chitosan nanoparticles (NPs) showed lower cell viability than gemcitabine solution at the highest concentration, 41.92 ± 2.01% and 26.23 ± 2.78%, respectively. Therefore, it was concluded that, along with the modified MTT method, these particles were safe to use in further cell culture studies.

CFPAC-1 cells were exposed to the increased concentrations of gemcitabine, empty, and gemcitabine-loaded NPs for up to 48 h. Ozturk et al. recommended a pH value of higher than 6.0 and a short incubation time of the blank chitosan and GEM-loaded chitosan nanoparticles to avoid aggregation of nanoparticles (Ozturk et al. 2020a). Considering the information in these data, we performed optimization experiments before determining our experimental setup. First, we treated CFPAC-1 cell lines with increased concentrations of chitosan nanoparticles and GEM-loaded chitosan nanoparticles for up to 72 h to measure pH values. We have found that up to 48 h, the pH value did not dramatically decrease in any NP-treated groups and stayed approximately at pH 7.0 (data not shown). Also, we prepared empty and GEM-loaded chitosan nanoparticles in serum-free media to minimize possible nanoparticle aggregations and performed our incubation for up to 48 h (Ozturk et al. 2020a). Morphological changes addressing gemcitabine-loaded NPs enhanced the anti-cancer properties of gemcitabine alone because we observed apoptotic cells and reduced cell proliferation in the gemcitabine-loaded NPs (Fig. 2) as reported previously (Ozturk et al. 2020b). We have supported this finding by evaluating G6PD and 6-PGD enzymes involved in cell proliferation via glycolysis and PPP. G6PD is the rate-limiting step of the PPP, mainly responsible for cell growth and development. Moreover, G6PD is accountable for NADPH production, an essential molecule for the function of antioxidant enzymes, including GR and GST (Aydemir et al. 2019a, b, 2020a). GR enzyme is mainly responsible for converting GSSG to GSH used by GST for maintaining cellular redox balance since an increased GSSG/GSH ratio is the primary biomarker for the increased oxidative stress in the cell or tissue. 6-PGD is another enzyme in the PPP pathway responsible for producing NADPH like G6PD (Aydemir et al. 2019a, b).

Our data showed that G6PD and 6-PGD enzyme activities significantly reduced in the gemcitabine and gemcitabine-loaded NP-administered cells compared to the control cells (Fig. 3). However, this reduction was enhanced in gemcitabine-loaded NP groups compared to the gemcitabine alone that data was supported by light microcopy pictures as well (Figs. 2 and 3). As mentioned before, G6PD and 6-PGD produce NADPH responsible for the homeostasis of the GSH/GSSG ratio. Our data showed that G6PD, 6-PGD, GR, GPx, and GST activities significantly reduced in the gemcitabine-loaded NP-administered groups compared to the control and GEM-treated cells at each concentration addressing decreased antioxidant response in the cell. However, gemcitabine alone induced antioxidant enzyme activities to reduce intrinsic oxidative stress (Fig. 3). Increased antioxidant response against GEM treatment is associated with chemoresistance and survival by avoiding oxidative stress in PDAC cells (Palam et al. 2015; Pan et al. 2021). On the other hand, oxidative stress is one of the significant regulators of transcription factor NF-KB involved in inflammation, immunity, and apoptosis. Our data showed that NF-KB levels significantly increased in GEM-loaded NPs at 1.32 and 2.64 µg/ml concentrations, controversially decreased in 0.66 and 5.32 µg/ml compared to the control and GEM treatment alone (Fig. 5) (Morgan and Liu 2011). GEM-loaded NPs can be promising tools to overcome chemoresistance and increase the sensitivity of tumors against GEM (Fig. 3).

Intrinsic apoptosis is regulated by a balance between anti-apoptotic (bcl-2, Bcl-xL, and Mcl-1) and pro-apoptotic (Bax, NOXA) proteins, the cell’s death life/death switch. Upregulation of bcl-2, Bcl-xL, and Mcl-1 and downregulation of Bax and NOXA lead to gemcitabine resistance in pancreatic cancer (Shi et al. 2002). Anti-apoptotic proteins Bcl-xL and NOXA/mcl-1 levels were lower in the cells treated with the gemcitabine-loaded NPs than in the gemcitabine-treated cells (Fig. 4) indicating activation of the intrinsic apoptotic pathway (Shi et al. 2002). MAPK, PI3K/Akt, JNK, STAT3, STAT5, ERK1/2, CREB, p38, and p70 are the constitutively activated pathways in PDAC associated with chemoresistance, metastasis, and progression (Tao et al. 2020). CREB, JNK, p38, p70, Akt, and STAT3 significantly reduced in GEM-loaded NPs and GEM-administered cells compared to the control cells at each concentration associated with enhanced apoptosis (Figs. 5 and 6) (Palam et al. 2015; Liu et al. 2022b). According to the literature, increased cytosolic Ca concentration is a hallmark of apoptosis, and cytosolic Ca^2+^ overload in PDAC triggers apoptosis (Richardson et al. 2020). On the other hand, glycolytic ATP regulates the plasma membrane calcium pumps to remove excessive intracellular Ca^2+^ to avoid apoptosis associated with GEM resistance in PDAC (James et al. 2013). Our data showed that GEM-loaded NPs significantly increased intracellular Ca^2+^ levels compared to the GEM alone and control cells indicating enhanced apoptosis (Fig. 7).

Ferroptosis is another mechanism contributing to the chemoresistance and metastasis in PDAC, regulated by oxidative stress, iron, and lipid metabolisms. Inducing ferroptosis, especially in iron-rich tissues such as the pancreas, has been discussed as a new strategy. Since ferroptosis is triggered via reduced antioxidant status, enhanced oxidative stress, iron and lipid peroxidation accumulation, enhanced glycolysis, and PPP inhibit ferroptosis via enhanced antioxidant status (Ping et al. 2022). Also, triggered ferroptosis has been correlated with the prognosis of PDAC, and the role of ferroptosis has not been widely studied (Yang et al. 2021). Increased oxidative stress, decreased antioxidant capacity, and increased Fe levels are markers of ferroptosis (Liu et al. 2021). GEM-loaded NPs significantly decreased antioxidant status in CFPAC-1 cells compared to the control and GEM-treated cells via decreasing G6PD, 6-PGD, GR, GST, and total GPx enzyme activities at each concentration (Fig. 3). In particular, GPX4 plays a significant role in the ferroptosis since GPX4 inhibits ferroptosis via inhibiting oxidative stress-induced lipid peroxidation (Chen et al. 2021). On the other hand, GR, GST, and GPX are glutathione-dependent enzymes, and inhibiting GSH-dependent antioxidant defenses triggers ferroptosis (Cao and Dixon 2016).

Another marker of ferroptosis is Fe involved in oxygen transport, hemoglobin metabolism, ATP production, oxidative stress metabolism, and DNA synthesis (Koppenol and Hider 2019). Increased intracellular Fe levels contribute to oxidative stress via Fenton reactions and lead to lipid peroxidation, which is critical for ferroptosis (Kruszewski 2003). Intracellular Fe levels significantly increased in GEM-loaded NP-administered groups compared to the GEM alone and control cells at each concentration, addressing enhanced apoptosis in CFPAC-1 cells (Fig. 7). On the other hand, Mn, Zn, and Mg are metals involved in the antioxidant enzymes, including Cu-Zn SOD, Mn-SOD, G6PD, and 6-PGD, responsible for maintaining antioxidant response in the cells. Therefore, increased concentrations of Cu, Zn, Mn, and Mg in the cell cause decreased antioxidant enzyme activity (Aydemir et al. 2022; Sahoo and Sharma 2023). Mg, Mn, and Zn levels significantly decreased in GEM-loaded NP-administered groups compared to the GEM alone and control cells at each concentration associated with reduced antioxidant status (Fig. 7).

The impact of ERK1/2 on cell survival and apoptosis is not very clear because, in some studies, ERK1/2 is correlated with gemcitabine resistance, whereas another paper suggested it can induce apoptosis as well (Wada and Penninger 2004). On the other hand, several studies indicated that ERK1/2 triggers oxidative stress-induced apoptosis and ferroptosis (Lee et al. 2003; Liu et al. 2022a). Our data showed that ERK1/2 levels significantly increased in GEM-loaded NPs at 1.32 and 2.64 µg/ml concentrations, controversially decreased in 0.66 and 5.32 µg/ml compared to the control and GEM treatment alone (Fig. 5). The role of the ERK1/2 in GEM-loaded NP-induced apoptosis and ferroptosis should be further investigated. In conclusion, for the first time in the literature, we have reported biocompatible gemcitabine-loaded NPs enhanced apoptosis and ferroptosis in CFPAC-1 cells harboring p53 and KRAS mutations compared to the GEM treatment alone. In vivo studies should be conducted to evaluate further whether GEM-loaded NPs trigger sensitivity against GEM treatment and overcome chemoresistance.

## Conclusion

GEM-based chemotherapy is a standard therapy for patients with PDAC, and 80% of all pancreatic cancer patients develop chemoresistance against GEM in locally advanced or metastatic phases, and the median survival of those patients is 6 months or less. Therefore, developing therapeutic approaches and increasing sensitivity to gemcitabine in PDAC has become one of the most significant challenges in cancer research. For the first time in the literature, we synthesized GEM-loaded NPs and evaluated their anti-cancer properties associated with oxidative stress, cellular toxicity, ferroptosis, glycolysis, and PPP metabolism using the CFPAC-1 cell line harboring KRAS and p53 mutations. GEM-loaded NPs enhanced ferroptosis and apoptosis via downregulation of PPP, glycolysis, and oxidative stress metabolism compared to the GEM alone. Our biocompatible GEM-loaded NPs are promising tools for overcoming GEM-related limitations and increasing the sensitivity of PDAC to GEM treatment. Further in vivo assays should be conducted to evaluate the anti-tumor activity of GEM-loaded NPs in animal models.

## Limitations of the study

In this study, we aim to show the anti-tumor effects of the GEM-loaded chitosan nanoparticles on the CFPAC-1 cells in comparison with GEM treatment alone in vitro since GEM causes side effects, poor overall survival, and 80% of patients often develop resistance rapidly to GEM. The first limitation of our study is that we performed our experimental setup in vitro because in vitro models do not fully represent the in vivo response. Effects of the GEM-loaded NPs cannot be thoroughly studied in the 2D cell cultures since they do not represent stroma in vivo. Also, metabolization, distribution, site targeting, and anti-cancer effects of the GEM-loaded nanoparticles can be revealed by in vivo experiments. Furthermore, peripheral organs should be investigated to assess the metabolization of the nanoparticles ***in vivo. ***After completing characterization and in vitro assays, we aimed to evaluate the anti-tumor effects of the GEM-loaded chitosan NPs in vivo compared with the GEM alone as a future aspect.

## Data Availability

No datasets were generated or analysed during the current study.

## References

[CR1] Aydemir D, Karabulut G, Şimşek G et al (2018) Impact of the Di(2-Ethylhexyl) Phthalate Administration on Trace element and Mineral levels in relation of kidney and liver damage in rats. Biol Trace Elem Res 186:474–488. 10.1007/s12011-018-1331-0 29654488 10.1007/s12011-018-1331-0

[CR2] Aydemir D, Hashemkhani M, Acar HY, Ulusu NN (2019a) In vitro interaction of glutathione S-transferase-pi enzyme with glutathione-coated silver sulfide quantum dots: a novel method for biodetection of glutathione S-transferase enzyme. Chem Biol Drug Des. 10.1111/cbdd.13614 10.1111/cbdd.13614 31452310

[CR3] Aydemir D, Hashemkhani M, Durmusoglu EG et al (2019b) A new substrate for glutathione reductase: glutathione coated Ag < Inf > 2 S quantum dots. Talanta 194. 10.1016/j.talanta.2018.10.049 10.1016/j.talanta.2018.10.049 30609564

[CR4] Aydemir D, Öztaşcı B, Barlas N, Ulusu NN (2019c) Effects of butylparaben on antioxidant enzyme activities and histopathological changes in rat tissues. Archives Industrial Hygiene Toxicol 70:315–324. 10.2478/aiht-2019-70-3342 10.2478/aiht-2019-70-3342 32623865

[CR5] Aydemir D, Sarayloo E, Ulusu NN (2019d) Rosiglitazone-induced changes in the oxidative stress metabolism and fatty acid composition in relation with trace element status in the primary adipocytes. J Med Biochem. 10.2478/jomb-2019-0041 10.2478/jomb-2019-0041 PMC7955996 33746608

[CR6] Aydemir D, Hashemkhani M, Acar HY, Ulusu NN (2020a) Evaluation of the biocompatibility of the GSH-coated Ag2S quantum dots in vitro: a perfect example for the non-toxic optical probes. Mol Biol Rep 47. 10.1007/s11033-020-05522-3 10.1007/s11033-020-05522-3 32436042

[CR7] Aydemir D, Simsek G, Ulusu NN (2020b) Dataset of the analyzing trace elements and minerals via ICP-MS: Method validation for the mammalian tissue and serum samples. Data Brief 29:105218. 10.1016/j.dib.2020.105218 10.1016/j.dib.2020.105218 PMC7016227 32071990

[CR8] Aydemir D, Surucu S, Basak AN, Ulusu NN (2022) Evaluation of the hematological and Serum Biochemistry Parameters in the pre-symptomatic and symptomatic stages of ALS Disease to support early diagnosis and prognosis. Cells 11:3569. 10.3390/cells11223569 36428998 10.3390/cells11223569 PMC9688239

[CR9] Barrera G, Cucci MA, Grattarola M et al (2021) Control of oxidative stress in cancer chemoresistance: spotlight on Nrf2 role. 10.3390/antiox10040510. Antioxidants 10:10.3390/antiox10040510 PMC8064392 33805928

[CR10] Bellich B, D’Agostino I, Semeraro S et al (2016) The Good, the bad and the Ugly of chitosans. Mar Drugs 14:99. 10.3390/md14050099 27196916 10.3390/md14050099 PMC4882573

[CR11] Cao JY, Dixon SJ (2016) Mechanisms of ferroptosis. Cell Mol Life Sci 73:2195–2209. 10.1007/s00018-016-2194-1 27048822 10.1007/s00018-016-2194-1 PMC4887533

[CR12] Chen X, Zeh HJ, Kang R et al (2021) Cell death in pancreatic cancer: from pathogenesis to therapy. Nat Rev Gastroenterol Hepatol 18:804–823. 10.1038/s41575-021-00486-6 34331036 10.1038/s41575-021-00486-6

[CR13] de Sousa Cavalcante L, Monteiro G (2014) Gemcitabine: metabolism and molecular mechanisms of action, sensitivity and chemoresistance in pancreatic cancer. Eur J Pharmacol 741. 10.1016/j.ejphar.2014.07.041 10.1016/j.ejphar.2014.07.041 25084222

[CR14] Fujiwara-Tani R, Sasaki T, Takagi T et al (2022) Gemcitabine Resistance in Pancreatic Ductal Carcinoma Cell Lines stems from reprogramming of Energy Metabolism. Int J Mol Sci 23:7824. 10.3390/ijms23147824 35887170 10.3390/ijms23147824 PMC9323155

[CR15] Garg U, Chauhan S, Nagaich U, Jain N (2019) Current advances in Chitosan nanoparticles Based Drug Delivery and Targeting. Adv Pharm Bull 9:195–204. 10.15171/apb.2019.023 31380245 10.15171/apb.2019.023 PMC6664124

[CR16] Halbrook CJ, Lyssiotis CA, Pasca di Magliano M, Maitra A (2023) Pancreatic cancer: advances and challenges. Cell 186:1729–1754. 10.1016/j.cell.2023.02.014 37059070 10.1016/j.cell.2023.02.014 PMC10182830

[CR17] Hamacher R, Schmid RM, Saur D, Schneider G (2008) Apoptotic pathways in pancreatic ductal adenocarcinoma. Mol Cancer 7. 10.1186/1476-4598-7-64 10.1186/1476-4598-7-64 PMC2515336 18652674

[CR18] James AD, Chan A, Erice O et al (2013) Glycolytic ATP fuels the plasma membrane calcium pump critical for pancreatic Cancer cell survival. J Biol Chem 288:36007–36019. 10.1074/jbc.M113.502948 24158437 10.1074/jbc.M113.502948 PMC3861649

[CR19] Ju H-Q, Gocho T, Aguilar M et al (2015) Mechanisms of overcoming intrinsic resistance to Gemcitabine in Pancreatic Ductal Adenocarcinoma through the Redox Modulation. Mol Cancer Ther 14:788–798. 10.1158/1535-7163.MCT-14-0420 25527634 10.1158/1535-7163.MCT-14-0420

[CR20] Khaira R, Sharma J, Saini V (2014) Development and characterization of nanoparticles for the delivery of gemcitabine hydrochloride. Sci World J 2014. 10.1155/2014/560962 10.1155/2014/560962 PMC3925564 24592173

[CR21] Koppenol WH, Hider RH (2019) Iron and redox cycling. Do’s and don’ts. Free Radic Biol Med 133:3–10. 10.1016/j.freeradbiomed.2018.09.022 30236787 10.1016/j.freeradbiomed.2018.09.022

[CR22] Kruszewski M (2003) Labile iron pool: the main determinant of cellular response to oxidative stress. Mutat Research/Fundamental Mol Mech Mutagen 531:81–92. 10.1016/j.mrfmmm.2003.08.004 10.1016/j.mrfmmm.2003.08.004 14637247

[CR23] Lee Y-J, Cho H-N, Soh J-W et al (2003) Oxidative stress-induced apoptosis is mediated by ERK1/2 phosphorylation. Exp Cell Res 291:251–266. 10.1016/S0014-4827(03)00391-4 14597424 10.1016/s0014-4827(03)00391-4

[CR24] Liu P, Wang Y, Li X (2019) Targeting the untargetable KRAS in cancer therapy. Acta Pharm Sin B 9:871–879. 10.1016/j.apsb.2019.03.002 31649840 10.1016/j.apsb.2019.03.002 PMC6804475

[CR25] Liu J, Kang R, Tang D (2021) The art of war: ferroptosis and pancreatic cancer. Front Pharmacol 12. 10.3389/fphar.2021.773909 10.3389/fphar.2021.773909 PMC8702849 34955844

[CR26] Liu N, Liang Y, Wei T et al (2022a) The role of ferroptosis mediated by NRF2/ERK-regulated ferritinophagy in CdTe QDs-induced inflammation in macrophage. J Hazard Mater 436:129043. 10.1016/j.jhazmat.2022.129043 10.1016/j.jhazmat.2022.129043 35525219

[CR27] Liu Y, Liu L, Zhang Y, Qin L (2022b) S-Adenosylmethionine enhances the inhibitory effect of gemcitabine against pancreatic cancer cells via suppression of the EGFR/AKT pathways. Mol Cell Toxicol 18:499–508. 10.1007/s13273-021-00220-y

[CR28] Malatesta M, Grecchi S, Chiesa E et al (2015) Internalized chitosan nanoparticles persist for long time in cultured cells. Eur J Histochem 59. 10.4081/ejh.2015.2492 10.4081/ejh.2015.2492 PMC4378219 25820565

[CR29] Morgan MJ, Liu Z (2011) Crosstalk of reactive oxygen species and NF-κB signaling. Cell Res 21:103–115. 10.1038/cr.2010.178 21187859 10.1038/cr.2010.178 PMC3193400

[CR30] Nilsen-Nygaard J, Strand S, Vårum K et al (2015) Chitosan: gels and Interfacial Properties. Polym (Basel) 7:552–579. 10.3390/polym7030552

[CR31] Otahal A, Aydemir D, Tomasich E, Minichsdorfer C (2020) Delineation of cell death mechanisms induced by synergistic effects of statins and erlotinib in non-small cell lung cancer cell (NSCLC) lines. Sci Rep 10:959. 10.1038/s41598-020-57707-2 31969600 10.1038/s41598-020-57707-2 PMC6976657

[CR32] Ozturk K, Arslan FB, Tavukcuoglu E et al (2020a) Aggregation of chitosan nanoparticles in cell culture: reasons and resolutions. Int J Pharm 578:119119. 10.1016/j.ijpharm.2020.119119 10.1016/j.ijpharm.2020.119119 32035256

[CR33] Ozturk K, Arslan FB, Tavukcuoglu E et al (2020b) Aggregation of chitosan nanoparticles in cell culture: reasons and resolutions. Int J Pharm 578. 10.1016/j.ijpharm.2020.119119 10.1016/j.ijpharm.2020.119119 32035256

[CR34] Öztürk K, Esendağlı G, Gürbüz MU et al (2017) Effective targeting of gemcitabine to pancreatic cancer through PEG-cored Flt-1 antibody-conjugated dendrimers. Int J Pharm 517. 10.1016/j.ijpharm.2016.12.009 10.1016/j.ijpharm.2016.12.009 27965135

[CR35] Palam LR, Gore J, Craven KE et al (2015) Integrated stress response is critical for gemcitabine resistance in pancreatic ductal adenocarcinoma. Cell Death Dis 6:e1913–e1913. 10.1038/cddis.2015.264 10.1038/cddis.2015.264 PMC4632294 26469962

[CR36] Pan R, Yuan Z, Liu Y et al (2021) A redox probe screens MTHFD1 as a determinant of gemcitabine chemoresistance in cholangiocarcinoma. Cell Death Discov 7:89. 10.1038/s41420-021-00476-2 33934113 10.1038/s41420-021-00476-2 PMC8088434

[CR37] Ping H, Jia X, Ke H (2022) A novel ferroptosis-related lncrnas signature predicts clinical prognosis and is associated with immune landscape in pancreatic cancer. Front Genet 13. 10.3389/fgene.2022.786689 10.3389/fgene.2022.786689 PMC8940287 35330729

[CR38] Quiñones JP, Peniche H, Peniche C (2018) Chitosan based self-assembled nanoparticles in drug delivery. Polymers (Basel) 10:. 10.3390/polym10030235 10.3390/polym10030235 PMC6414940 30966270

[CR39] Richardson DA, Sritangos P, James AD et al (2020) Metabolic regulation of calcium pumps in pancreatic cancer: role of phosphofructokinase-fructose-bisphosphatase-3 (PFKFB3). Cancer Metab 8:2. 10.1186/s40170-020-0210-2 10.1186/s40170-020-0210-2 PMC7114799 32266066

[CR40] Sahoo K, Sharma A (2023) Understanding the mechanistic roles of environmental heavy metal stressors in regulating ferroptosis: adding new paradigms to the links with diseases. Apoptosis 28:277–292. 10.1007/s10495-022-01806-0 36611106 10.1007/s10495-022-01806-0

[CR41] Shi X, Liu S, Kleeff J et al (2002) Acquired resistance of pancreatic cancer cells towards 5-fluorouracil and gemcitabine is associated with altered expression of apoptosis-regulating genes. 10.1159/000065068. Oncology 62:10.1159/000065068 12138244

[CR42] Singla AK, Chawla M (2010) Chitosan: some pharmaceutical and biological aspects - an update. J Pharm Pharmacol 53:1047–1067. 10.1211/0022357011776441 10.1211/0022357011776441 11518015

[CR43] Sonveaux P, Végran F, Schroeder T et al (2008) Targeting lactate-fueled respiration selectively kills hypoxic tumor cells in mice. J Clin Invest. 10.1172/JCI36843 10.1172/JCI36843 PMC2582933 19033663

[CR44] Sun Y, Ren D, Zhou Y et al (2021) Histone acetyltransferase 1 promotes gemcitabine resistance by regulating the PVT1/EZH2 complex in pancreatic cancer. Cell Death Dis 12. 10.1038/s41419-021-04118-4 10.1038/s41419-021-04118-4 PMC8464605 34564701

[CR45] Tao H, Chen X, Du Z, Ding K (2020) Corn silk crude polysaccharide exerts anti-pancreatic cancer activity by blocking the EGFR/PI3K/AKT/CREB signaling pathway. Food Funct 11:6961–6970. 10.1039/D0FO00403K 32696775 10.1039/d0fo00403k

[CR46] Wada T, Penninger JM (2004) Mitogen-activated protein kinases in apoptosis regulation. Oncogene 23. 10.1038/sj.onc.1207556 10.1038/sj.onc.1207556 15077147

[CR47] Yang Y, Zhang Z-J, Wen Y et al (2021) Novel perspective in pancreatic cancer therapy: targeting ferroptosis pathway. World J Gastrointest Oncol 13:1668–1679. 10.4251/wjgo.v13.i11.1668 34853642 10.4251/wjgo.v13.i11.1668 PMC8603450

[CR48] Zhang E, Xing R, Liu S et al (2019) Advances in chitosan-based nanoparticles for oncotherapy. Carbohydr Polym 222. 10.1016/j.carbpol.2019.115004 10.1016/j.carbpol.2019.115004 31320066

